# Occupation and prostate Cancer risk: results from the epidemiological study of prostate cancer (EPICAP)

**DOI:** 10.1186/s12995-022-00346-2

**Published:** 2022-02-07

**Authors:** Wendy Bijoux, Emilie Cordina-Duverger, Soumaya Balbolia, Pierre-Jean Lamy, Xavier Rebillard, Brigitte Tretarre, Sylvie Cenee, Florence Menegaux

**Affiliations:** 1 Université Paris-Saclay, UVSQ, CESP (Center for Research in Epidemiology and Population Health), Inserm, Villejuif, France; 2 Institut Gustave Roussy, Villejuif, France; 3 Institut médical d’Analyse Génomique-Imagenome, Labosud, Montpellier, France; 4 Service Urologie, Clinique Beau Soleil, Montpellier, France; 5 Registre des Tumeurs de l’Hérault, EA 2415 Montpellier, ICM France

**Keywords:** Prostate cancer, Aggressive prostate cancer, Occupation, Case-control study

## Abstract

**Background:**

Although prostate cancer (PCa) is the most frequent male cancer in industrialized countries, little is known about its aetiology. The literature has suggested an influence of the environment, including occupational exposures, but results are inconsistent. In this context, we investigated PCa risk associated to employment among several occupations using data from EPICAP study.

**Methods:**

EPICAP is a French population-based case-control study including 819 PCa incident cases and 879 controls frequency-matched on age. In-person interviews gathered data on potential risk factors and lifetime occupational histories for each job held at least 6 months. Then, occupations were coded using ISCO 68. Unconditional logistic regressions were performed to assess the association between occupations (ever occupied and by duration) and PCa risk, whether all and aggressive, after adjusting for potential confounders.

**Results:**

For ≥10 years of employment, we found positive associations with PCa, whether overall and aggressive, among Medical, Dental and Veterinary workers (OR (odds ratios) =5.01 [95% confidence interval] [1.27; 19.77]), Members of the armed forces (OR = 5.14 [0.99; 26.71]) and Fishermen, hunters and related workers (OR = 4.58 [1.33; 15.78]); whether overall and non-aggressive PCa, among Legislative officials and Government administrators (OR = 3.30 [1.10; 9.84]) or Managers (OR = 1.68 [1.18; 2.41]); however a negative association, whether overall and non-aggressive PCa, among Material-Handling and Related Equipment Operators, Dockers and Freight Handlers (OR = 0.40 [0.17; 0.97]).

**Conclusion:**

Excess PCa risks were observed in the EPICAP study mostly among white collar workers exposed to several factors in their work environment. These emerging associations can be used to lead future research investigating specific occupational exposures.

**Supplementary Information:**

The online version contains supplementary material available at 10.1186/s12995-022-00346-2.

## Background

Prostate cancer (PCa) is the first male cancer in industrialized countries, including France [1]. In 2020, based on GLOBOCAN estimates, 1,276,106 new cases were registered over the world, 449,761 in Europe with 64,955 in France. Meanwhile, 358,989 men worldwide, 107,315 in Europe with 9002 in France died from this cancer [2, 3].

Numerous studies in the past decades focused on identifying the aetiology of PCa, however, it remains largely unknown. The only recognized risk factors are advanced age, ethnic origin and family history of PCa. Epidemiological studies on migrants showed an increase of the incidence of PCa for Asians living in the United states compared to those living in their native countries, suggesting the role of lifestyle and environmental factors, including occupational factors [4].

Few original studies have focused on the influence of occupational factors on the occurrence of PCa before the 90s. To date, we identified 5 cohorts [5–10] and 10 case-control studies [11–20], two literature reviews [21–23] and two meta-analysis [24, 25] investigating the role of occupational factors on PCa risk. Among these, the risk of PCa was evaluated, yet with inconclusive results, for farmers [5, 6, 15, 16, 19, 21, 22, 24], pesticide applicators and manufacturers [10, 21, 22, 24], protective service workers [5–9, 11, 14–17, 21], administrative and managerial workers [8–11, 13, 14, 16–19, 22], workers with jobs related to low physical activity [12, 16, 22, 24], night shift workers [23, 25, 26], heavy and toxic metals and chemical workers [5, 7, 14, 18, 21, 22, 24, 27, 28].

However, several studies presented methodological weaknesses particularly in occupational exposure assessment or study design: studies focusing on specific occupational groups, few studies in general population, few studies with a sufficient statistical power to go deeply in the analyses, studies using current or longest job titles as proxies of lifetime occupational exposures [7, 8, 20] or studies that did not take into account potential confounding factors [19, 20]. Moreover, only two studies were able to specifically address the aggressiveness of PCa [11, 15].

In this context, we aimed to investigate PCa risk associated to employment among several occupations, taking into account PCa aggressiveness, using data from EPICAP study.

## Methods

### Study population

EPICAP (Epidemiological study of prostate cancer) is a population-based case-control study conducted in the department of Hérault between 2012 and 2014 to specifically address the role of environmental, occupational and genetic factors on the occurrence of PCa. Details of the study design have been described elsewhere [29].

Briefly, eligible cases were all male newly diagnosed with PCa in 2012–2013, less than 75 years old, living in Hérault at diagnosis, histologically confirmed cancer cases recruited in all public and private centres of the geographic area. Hérault is located in the southeast of the country. This department was chosen for the existence of a departmental cancer registry created more than 26 years ago, its mixed agricultural and urban character, and its large size (more than 1 million inhabitants). In 2011, the Hérault Cancer Registry observed 770 new cases of prostate cancer, of which 575 were under 75 years of age. Considering that the number of cases in 2012–2013 will be similar, approximately 1150 new cases were expected during the study period (2012–2013). In fact, 1098 eligible cases were identified over the study period suggesting that the identification of cases in the EPICAP study was exhaustive.

Eligible controls were men selected among the general population living in the department of Hérault, free from this cancer, and frequency-matched on age (5 year age group) with cases. Quotas for age and socio-economic status (SES) were defined in order to minimize selection bias. Thus, the age distribution of the selected controls had to reflect the age distribution observed among the cancer cases. In addition, the distribution of the control group had to reflect the SES distribution of the male population of the same age in the department of Hérault, based on data from the population census given by The National Institute of Statistics and Economic Studies (INSEE). Phone numbers of private homes, obtained via a survey institute (IPSOS), were selected at random from the telephone directory of the Hérault department. In order to maximize the possibility to reach the households, they were called up to 15 times at different hours and days during week-days and week-ends. If the household contained a male who met the eligibility criteria, he was invited to participate to the study as long as the predefined quota corresponding to his age group and SES was not reached.

Finally, the study included 819 incident cases and 879 controls with a participation rate of 75 and 79% respectively. Each subject included in EPICAP signed a written informed consent.

### Data collection

All participants were face-to-face interviewed by a trained research clinical nurse, using a standardised computer assisted questionnaire. Information related to usual sociodemographic characteristics, personal and family medical history, hormonal and metabolic factors, infectious and inflammatory factors, lifestyle factors and environmental factors including complete residence and occupational history were collected.

Cases and controls went through anthropomorphic measurements during the interview and blood samples or saliva samples were proposed to both of them.

Clinical data of all cases were gathered from medical record of each case including prostatic specific antigen (PSA) levels and Gleason Score at diagnosis and validated by the Hérault cancer registry.

### Coding of occupation titles

Participants answered a lifetime occupational questionnaire covering all jobs held more than 6 months. For each job held, information on starting and ending dates, name and address of the company, description of the tasks involved were gathered. For some specific jobs, a more detailed questionnaire was answered by participants. Then, an industrial hygienist coded the job titles blinded to the subject’s case/control status using the International Standard Classification of Occupations (ISCO) 1968. ISCO’s structure uses a decimal method of coding and has four levels, providing successively finer detail about each occupation: 1-digit major groups, 2-digit minor groups, 3-digit unit groups and 5-digit occupational categories [30]. In the present study, we considered 8 major, 61 minor and 99 unit groups for occupational categories with at least ten subjects ever employed. For each category, we used two indicators: ever employment (no, yes) and lifetime duration of employment (< 10 years, ≥10 years).

### Statistical analysis

Since one case and four controls did not fill the occupational questionnaire, the analyses were restricted to 818 cases and 875 controls and performed with the statistical analysis software SAS (9.4 version).

For a given occupational group, associations between occupation and PCa were studied using unconditional logistic regression models which calculated odds ratios (OR) and 95% confidence intervals [95% CI]. The reference group for each occupational category was the men never employed in that particular occupation. The analyses were systematically adjusted for the well-established risk factors: age, ethnic origin, and first-degree family history of PCa. Our final models were also adjusted for other potential confounding factors such as marital status, waist circumference and physical activity that were different between cases and controls with a *p*-value less than 0.2. Educational level, highly related to screening behaviour, was also included in our final models.

We also performed multinomial logistic regression models to evaluate associations according to PCa aggressiveness using the Gleason score at diagnosis. If Gleason Score was < 7 or equivalent to 7 (3 + 4), it was considered as non-aggressive cancer and if it was equivalent to 7 (4 + 3) or ≥ 8, then it was considered as aggressive PCa [31]. Since the Gleason score was missing for 13 cases, the analyses by prostate cancer aggressiveness was based on 1680 participants.

## Results

Characteristics of the EPICAP population are presented in Table 1. Among cases, 623 (77.4%) were classified as non-aggressive cancer and 182 (22.6%) as aggressive PCa. Among the 806 controls who provided information on prostate cancer screening, 606 (75.2%) were screened in the last 2 years before the interview, 66 (8.2%) more than 2 years before interview, and 134 (16.6) had never been screened.

**Table 1:** Selected characteristics of the EPICAP study population (*N* = 1693)

Characteristics	Cases ***n*** = 818 (%)	Controls ***n*** = 875 (%)	***p***-value*
**Gleason score at diagnosis**			
≤ 7^†^	623 (77.4)	–	
≥ 7^††^	182 (22.6)	–	
**Age**			*0.150*
< 55 years	48 (5.9)	59 (6.7)	
55–59 years	99 (12.1)	99 (11.3)	
60–64 years	216 (26.4)	200 (22.9)	
65–69 years	274 (33.5)	283 (32.3)	
≥ 70 years	181 (22.1)	234 (26.8)	
**Ethnic origin**			*0.402*
Caucasian,	794 (97.1)	855 (97.7)	
Others	24 (2.9)	20 (2.3)	
**First-degree family history of prostate cancer**			***< 0.001***
no	548 (75.2)	722 (90.5)	
yes	181 (24.8)	76 (9.5)	
**Educational level**			*0.503*
none	70 (8.5)	71 (8.1)	
primary school	376 (46.0)	434 (49.7)	
high school	112 (13.7)	109 (12.5)	
university	260 (31.8)	260 (29.7)	
**Marital status**			*0.193*
married/domestic partnership	674 (82.4)	748 (85.5)	
divorced/separated/single	115 (14.1)	98 (11.2)	
widowed	29 (3.5)	29 (3.3)	
**Smoking status**			*0.362*
non-smokers	240 (29.4)	246 (28.1)	
former smokers	454 (55.6)	475 (54.3)	
current smokers	123 (15.0)	154 (17.6)	
**Regular alcohol drinking**			*0.576*
no	72 (8.8)	84 (9.6)	
yes	744 (91.2)	790 (90.4)	
**Intensity of physical activity**			*0.123*
< 1 h/week	191 (23.5)	177 (20.3)	
lifetime MET between 0.10–6.23	153 (18.8)	172 (19.8)	
lifetime MET between 6.25–13.01	133 (16.4)	172 (19.8)	
lifetime MET between 13.07–24.11	149 (18.3)	175 (20.1)	
lifetime MET between 24.15–351.40	187 (23.0)	174 (20.0)	
**Waist circumference (in centimetres)**			*0.082*
≤ 94	208 (25.8)	253 (29.6)	
> 94	599 (74.2)	602 (70.4)	
**Body-mass index 2 years before diagnosis or interview, in kg/m**^**2**^			*0.862*
< 25	296 (36.7)	314 (36.5)	
25–29	377 (46.7)	394 (45.9)	
≥ 30	134 (16.6)	151 (17.6)	
**Night shift work**			*0.522*
never	532 (65.0)	556 (63.5)	
ever	286 (35.0)	319 (36.5)	
**Last screening for prostate cancer before interview, in years**			
≤ 2	–	606 (75.2)	
> 2	–	66 (8.2)	
Never screened	–	134 (16.6)	

**Abbreviations.** *p-value: Chi-2 test; ^†^ ≤ 7(3 + 4): low aggressive cancer; ^††^ ≥ 7 (4 + 3): high aggressive cancer; *MET* Metabolic Equivalent of Task

Beside first-degree family history of PCa, which was significantly different between cases and controls (*p < 0.001*), all other characteristics of the study population were comparable between cases and controls.

Regarding lifetime occupational history of the 1693 participants, a total of 11,622 jobs have been held for more than 6 months. The average number of jobs held per men during lifetime was 5 jobs (range 1 to 27).

In Table 2 and 3, we reported the results for all the major groups (1-digit) and only minor groups (2-digits) and unit groups (3-digits) with at least one statistically significant association for ever employment or duration of employment with overall PCa or PCa aggressiveness. The overall results can be found in the Additional file 1 (Table S1 for overall PCa and S2 for PCa aggressiveness).

**Table 2:** Associations between selected occupations and prostate cancer [all cancers] (*N* = 1693)

ISCO^*^ Code (1 to 3 digits): Description	Never	Ever employed		< 10 years employed		≥ 10 years employed	
	Ca/Co^**^	Ca/Co	OR [95% CI]^†^	Ca/Co	OR [95% CI]	Ca/Co	OR [95% CI]
***0/1: PROFESSIONAL, TECHNICAL AND RELATED WORKERS***	516/571	302/304	1.10 [0.86; 1.40]	88/102	1.00 [0.72; 1.40]	214/202	1.16 [0.87; 1.53]
**0–2: Architects, Engineers**	785/836	33/39	0.83 [0.50; 1.39]	9/21	**0.43 [0.19; 0.96]**	24/18	1.32 [0.69; 2.54]
**0–3: Surveyors, Draughtsmen and Related technicians**	735/804	83/71	1.34 [0.95; 1.90]	37/41	1.06 [0.66; 1.70]	46/30	**1.72 [1.06; 2.81]**
**0–6: Medical, Dental and Veterinary workers**	799/869	19/6	**3.21 [1.24; 8.34]**	1/1	0.80 [0.05; 13.88]	18/5	**3.75 [1.34; 10.49]**
0–61: Medical Doctors	807/872	11/3	**3.98 [1.08; 14.72]**	0/0	–	11/3	**3.98 [1.08; 14.72]**
***2: ADMINISTRATIVE AND MANAGERIAL WORKERS***	671/760	147/115	**1.47 [1.10; 1.97]**	29/35	0.92 [0.54; 1.58]	118/80	**1.72 [1.24; 2.38]**
**2–0: Legislative Officials and Government Administrators**	796/865	22/10	**2.33 [1.07; 5.07]**	10/5	1.83 [0.61; 5.50]	12/5	2.88 [0.98; 8.41]
2–02: Government Administrators	798/867	20/8	**2.80 [1.20; 6.52]**	8/3	2.66 [0.69; 10.27]	12/5	**2.88 [0.99; 8.43]**
**2–1: Managers**	685/767	133/108	**1.39 [1.03; 1.86]**	28/34	0.89 [0.52; 1.54]	105/74	**1.62 [1.15; 2.27]**
2–11: General Managers	769/843	49/32	**2.03 [1.24; 3.31]**	15/13	1.58 [0.70; 3.57]	34/19	**2.31 [1.26; 4.23]**
***3: CLERICAL AND RELATED WORKERS***	580/611	238/264	0.91 [0.73; 1.14]	102/100	1.01 [0.73; 1.39]	136/164	0.85 [0.65; 1.11]
***4: SALES WORKERS***	618/693	200/182	1.23 [0.97; 1.56]	97/89	1.16 [0.84; 1.61]	103/93	1.29 [0.94; 1.78]
**4–0: Managers (Wholesale and Retail Trade)**	796/864	22/11	2.00 [0.94; 4.27]	12/3	3.31 [0.88; 12.42]	10/8	1.49 [0.58; 3.85]
***5: SERVICE WORKERS***	681/700	137/175	0.78 [0.60; 1.01]	56/94	**0.55 [0.38; 0.80]**	81/81	1.05 [0.75; 1.48]
**5–5: Building Caretakers, Charworkers, Cleaners and Related Workers**	806/849	12/26	**0.44 [0.21; 0.90]**	10/19	0.49 [0.22; 1.10]	2/7	0.28 [0.05; 1.47]
**5–8: Protective Service Workers**	762/805	56/70	0.81 [0.55; 1.19]	21/34	0.60 [0.33; 1.06]	35/36	1.02 [0.62; 1.67]
***6: AGRICULTURAL ANIMAL HUSBANDRY AND FORESTRY WORKERS, FISHERMEN AND HUNTERS***	707/762	111/113	1.00 [0.74; 1.35]	49/54	0.92 [0.60; 1.40]	62/59	1.07 [0.72; 1.59]
**6–4: Fishermen, Hunters and Related Workers**	803/867	15/8	1.96 [0.81; 4.76]	2/2	1.14 [0.15; 8.48]	13/6	2.23 [0.82; 6.06]
6–41: Fishermen	809/870	9/5	1.93 [0.62; 5.97]	1/1	1.29 [0.08; 21.33]	8/4	2.08 [0.60; 7.21]
***7/8/9: PRODUCTION AND RELATED WORKERS, TRANSPORT EQUIPMENT OPERATORS AND LABOURERS***	395/364	423/511	**0.76 [0.60; 0.95]**	136/160	0.78 [0.58; 1.04]	287/351	**0.74 [0.58; 0.96]**
**7–0: Production Supervisors and General Foremen**	747/821	71/54	**1.60 [1.09; 2.34]**	28/24	1.36 [0.76; 2.44]	43/30	**1.78 [1.09; 2.91]**
**7–4: Chemical Processers and Related Workers**	817/866	1/9	**0.11 [0.01; 0.86]**	0/5	–	1/4	0.26 [0.03; 2.50]
**7–7: Food and Beverage Processers**	789/828	29/47	**0.58 [0.35; 0.96]**	16/25	0.59 [0.30; 1.15]	13/22	0.58 [0.28; 1.19]
**8–1: Cabinetmakers and Related Woodworkers**	804/870	14/5	**2.94 [1.03; 8.43]**	9/2	4.03 [0.84; 19.33]	5/3	2.16 [0.50; 9.29]
**8–3: Blacksmiths, Toolmakers and Machine Tool Operators**	785/820	33/55	0.69 [0.43; 1.09]	16/34	**0.53 [0.28; 0.98]**	17/21	0.96 [0.49; 1.88]
8–39: Blacksmiths, Toolmakers and Machine-Tool Operators Not Elsewhere Classified	808/857	10/18	0.66 [0.29; 1.51]	1/14	**0.09 [0.01; 0.68]**	9/4	2.58 [0.76; 8.72]
**9–7: Material Handling and Related Equipment Operators, Dockers and Freight Handlers**	760/794	58/81	0.74 [0.51; 1.08]	48/58	0.86 [0.56; 1.32]	10/23	**0.44 [0.20; 0.95]**
**9–8: Transport Equipment Operators**	730/752	88/123	**0.73 [0.53; 0.99]**	37/63	**0.61 [0.39; 0.94]**	51/60	0.85 [0.56; 1.29]
9–85: Motor vehicle drivers	747/780	71/95	0.77 [0.54; 1.09]	31/48	**0.55 [0.32; 0.95]**	40/47	0.95 [0.58; 1.53]
***MEMBERS OF THE ARMED FORCES***	802/857	16/18	0.97 [0.48; 1.94]	10/15	0.72 [0.32; 1.65]	6/3	2.21 [0.53; 9.12]

*Abbreviations*: ^+^occupations (exercised by 10 subjects or more) with at least one statistically significant association for ever employment or duration of employment; ^*^ISCO: international standard classification of occupations (version of 1968); ^**^Ca/Co: cases/controls; ^†^Odds ratio adjusted for age (age of reference (60–64 years)), ethnic origin, first-degree family history of this cancer, educational level, intensity of physical activity, waist circumference, marital status; ^***^Other health professionals: Nurses, Midwives, Optometrists and opticians, Physiotherapists and occupational therapists, Medical radiology technicians

**Table 3:** Associations between selected occupations^+^ and prostate cancer aggressiveness (*N* = 1680)

ISCO^*^ Code (1 to 3 digits): Description	CA^**^	Never	Ever employed		< 10 years employed		≥ 10 years employed	
		n^***^	n	OR [95% CI]^****^	n	OR [95% CI]	n	OR [95% CI]
***0/1: PROFESSIONAL, TECHNICAL AND RELATED WORKERS***	NA^†^ A^††^ C^†††^	401 106 571	222 76 304	0.96 [0.73; 1.25] **1.63 [1.11; 2.42]**	64 22 102	0.89 [0.61; 1.28] 1.36 [0.79; 2.32]	158 54 202	1.00 [0.73; 1.35] **1.83 [1.17; 2.86]**
**0–2: Architects, Engineers**	NA A C	595 177 836	28 5 39	0.89 [0.52; 1.52] 0.68 [0.25; 1.80]	8 1 21	0.48 [0.20; 1.12] 0.24 [0.03; 1.87]	20 4 18	1.38 [0.69; 2.75] 1.18 [0.38; 3.66]
**0–3: Surveyors, Draughtsmen and Related technicians**	NA A C	565 158 804	58 24 71	1.23 [0.85; 1.80] **1.68 [1.00; 2.81]**	27 10 41	0.99 [0.59; 1.67] 1.35 [0.65; 2.79]	31 14 30	1.56 [0.92; 2.65] **2.11 [1.06; 4.21]**
**0–6: Medical, Dental and Veterinary workers**	NA A C	609 177 869	14 5 6	**2.90 [1.07; 7.89]** **4.63 [1.33; 16.15]**	0 1 1	- 2.01 [0.10; 39.56]	14 4 5	**3.53 [1.22; 10.22]** **5.01 [1.27; 19.77]**
0–61: Medical Doctors	NA A C	614 180 872	9 2 3	**3.94 [1.03; 15.04]** 4.16 [0.65; 26.55]	0 0 0	- -	9 2 3	**3.94 [1.03; 15.04]** 4.16 [0.65; 26.55]
***2: ADMINISTRATIVE AND MANAGERIAL WORKERS***	NA A C	507 154 760	116 28 115	**1.51 [1.11; 2.06]** 1.29 [0.80; 2.06]	21 8 35	0.83 [0.46; 1.51] 1.28 [0.57; 2.91]	95 20 80	**1.82 [1.29; 2.57]** 1.30 [0.75; 2.23]
**2–0: Legislative Officials and Government Administrators**	NA A C	604 180 865	19 2 10	**2.52 [1.13; 5.62]** 1.06 [0.22; 5.03]	8 1 5	1.83 [0.58; 5.79] 0.84 [0.10; 7.49]	11 1 5	**3.30 [1.10; 9.84]** 1.29 [0.15; 11.41]
2–02: Government Administrators	NA A C	606 180 867	17 2 8	**3.00 [1.25; 7.19]** 1.38 [0.28; 6.74]	6 1 3	2.55 [0.62; 10.52] 1.42 [0.14; 14.23]	11 1 5	**3.30 [1.11; 9.85]** 1.30 [0.15; 11.49]
**2–1: Managers**	NA A C	520 155 767	103 27 108	**1.39 [1.02; 1.91]** 1.30 [0.81; 2.10]	20 8 34	0.79 [0.43; 1.45] 1.31 [0.58; 2.96]	83 19 74	**1.68 [1.18; 2.41]** 1.31 [0.75; 2.28]
2–11: General Managers	NA A C	586 172 843	37 10 32	**2.01 [1.19; 3.38]** 1.82 [0.86; 3.89]	10 4 13	1.31 [0.53; 3.24] 2.12 [0.65; 6.95]	27 6 19	**2.45 [1.30; 4.63]** 1.70 [0.65; 4.45]
***3: CLERICAL AND RELATED WORKERS***	NA A C	439 132 611	184 50 264	0.94 [0.74; 1.20] 0.82 [0.56; 1.19]	81 20 100	1.08 [0.77; 1.51] 0.84 [0.49; 1.44]	103 30 64	0.86 [0.64; 1.15] 0.81 [0.52; 1.26]
***4: SALES WORKERS***	NA A C	465 144 693	158 38 182	**1.30 [1.00; 1.68]** 1.01 [0.67; 1.51]	72 23 89	1.13 [0.80; 1.61] 1.24 [0.75; 2.06]	86 15 93	**1.47 [1.05; 2.05]** 0.78 [0.43; 1.40]
**4–0: Managers (Wholesale and Retail Trade)**	NA A C	607 177 864	16 5 11	1.86 [0.83; 4.20] 1.95 [0.65; 5.80]	7 4 3	2.12 [0.50; 8.98] **5.66 [1.19; 26.83]**	9 1 8	1.86 [0.70; 4.96] 0.54 [0.07; 4.43]
***5: SERVICE WORKERS***	NA A C	516 153 700	107 29 175	0.81 [0.61; 1.07] 0.72 [0.46; 1.13]	43 12 94	**0.55 [0.37; 0.83]** 0.55 [0.29; 1.03]	64 17 81	1.12 [0.78; 1.62] 0.94 [0.53; 1.65]
**5–5: Building Caretakers, Charworkers, Cleaners and Related Workers**	NA A C	613 180 849	10 2 26	0.47 [0.22; 1.02] 0.35 [0.08; 1.49]	8 2 19	0.50 [0.21; 1.23] 0.49 [0.11; 2.16]	2 0 7	0.38 [0.07; 2.03] -
**5–8: Protective Service Workers**	NA A C	579 171 805	44 11 70	0.84 [0.56; 1.27] 0.70 [0.36; 1.37]	16 4 34	0.58 [0.31; 1.10] 0.52 [0.18; 1.52]	28 7 36	1.09 [0.64; 1.84] 0.87 [0.38; 2.03]
***6: AGRICULTURAL ANIMAL HUSBANDRY AND FORESTRY WORKERS, FISHERMEN AND HUNTERS***	NA A C	547 150 762	76 32 113	0.89 [0.64; 1.24] 1.30 [0.83; 2.05]	35 13 54	0.85 [0.53; 1.35] 1.15 [0.60; 2.20]	41 19 59	0.93 [0.60; 1.44] 1.44 [0.81; 2.57]
**6–4: Fishermen, Hunters and Related Workers**	NA A C	614 177 867	9 5 8	1.53 [0.57; 4.11] **3.16 [1.00; 10.05]**	2 0 2	1.66 [0.22; 12.50] -	7 5 6	1.54 [0.50; 4.74] **4.58 [1.33; 15.78]**
6–41: Fishermen	NA A C	619 178 870	4 4 5	1.13 [0.29; 4.39] **4.01 [1.02; 15.69]**	1 0 1	1.84 [0.11; 30.43] -	3 4 4	1.01 [0.22; 4.75] **5.10 [1.20; 21.61]**
***7/8/9: PRODUCTION AND RELATED WORKERS, TRANSPORT EQUIPMENT OPERATORS AND LABOURERS***	NA A C	305 86 364	318 96 511	**0.76 [0.59; 0.97]** 0.73 [0.50; 1.06]	107 26 160	0.81 [0.59; 1.12] 0.63 [0.38; 1.04]	211 70 351	**0.72 [0.55; 0.95]** 0.79 [0.52; 1.18]
**7–0: Production Supervisors and General Foremen**	NA A C	570 166 821	53 16 54	**1.60 [1.06; 2.41]** 1.60 [0.88; 2.89]	22 5 24	1.43 [0.77; 2.65] 1.11 [0.41; 3.02]	31 11 30	**1.73 [1.02; 2.94]** 1.98 [0.96; 4.09]
**7–4: Chemical Processers and Related Workers**	NA A C	622 182 866	1 0 9	0.14 [0.02; 1.18] -	0 0 5	- -	1 0 4	0.37 [0.04; 3.57] -
**7–7: Food and Beverage Processers**	NA A C	600 176 828	23 6 47	0.62 [0.36; 1.07] 0.51 [0.21; 1.23]	11 5 25	0.54 [0.25; 1.16] 0.76 [0.28; 2.08]	12 1 22	0.72 [0.34; 1.51] 0.19 [0.03; 1.46]
**8–1: Cabinetmakers and Related Woodworkers**	NA A C	611 180 870	12 2 5	**3.53 [1.20; 10.39** 1.62 [0.30; 8.65]	8 1 2	**4.97 [1.01; 24.42]** 1.84 [0.16; 21.03]	4 1 3	2.46 [0.53; 11.30] 1.52 [0.15; 15.20]
**8–3: Blacksmiths, Toolmakers and Machine Tool Operators**	NA A C	599 173 820	24 9 55	0.67 [0.40; 1.12] 0.83 [0.40; 1.74]	12 4 34	0.53 [0.27; 1.05] 0.57 [0.20; 1.66]	12 5 21	0.90 [0.43; 1.90] 1.29 [0.47; 3.55]
8–39: Blacksmiths, Toolmakers and Machine-Tool Operators Not Elsewhere Classified	NA A C	617 178 857	6 4 18	0.52 [0.20; 1.37] 1.19 [0.39; 3.66]	1 0 14	**0.12 [0.02; 0.92]** -	5 4 4	1.83 [0.47; 7.19] 5.36 [1.28; 22.45]
**9–7: Material Handling and Related Equipment Operators, Dockers and Freight Handlers**	NA A C	585 162 794	38 20 81	**0.62 [0.41; 0.95]** 1.24 [0.72; 2.15]	31 17 58	0.71 [0.44; 1.15] 1.51 [0.83; 2.75]	7 3 23	**0.40 [0.17; 0.97]** 0.61 [0.18; 2.09]
**9–8: Transport Equipment Operators**	NA A C	558 159 752	65 23 123	**0.71 [0.50; 1.00]** 0.85 [0.52; 1.40]	30 7 63	0.65 [0.41; 1.05] 0.51 [0.22; 1.14]	35 16 60	0.76 [0.48; 1.21] 1.22 [0.67; 2.24]
9–85: Motor-Vehicle Drivers	NA A C	571 163 780	52 19 95	0.74 [0.50; 1.08] 0.93 [0.54; 1.60]	25 6 48	0.68 [0.40; 1.15] 0.57 [0.24; 1.39]	27 13 47	0.80 [0.48; 1.34] 1.31 [0.67; 2.55]
***MEMBERS OF THE ARMED FORCES***	NA A C	612 177 857	11 5 18	0.87 [0.40; 1.90] 1.36 [0.49; 3.77]	8 2 15	0.76 [0.31; 1.84] 0.64 [0.14; 2.88]	3 3 3	1.46 [0.29; 7.48] **5.14 [0.99; 26.71]**

*Abbreviations*: ^+^occupations (exercised by 10 subjects or more) with at least one statistically significant association for ever employment or duration of employment; ^*^ISCO: international standard classification of occupations (version of 1968); ^**^CA: cancer aggressiveness; ^***^n: number of participants in each group; ^****^Odds ratio adjusted for age (age of reference (60–64 years)), ethnic origin, first-degree family history of this cancer, educational level, intensity of physical activity, waist circumference, marital status; ^†^NA: non-aggressive cancer cases (Gleason score ≥ 7 (3 + 4)); ^††^A: aggressive cancer cases (Gleason score ≤ 7 (4 + 3)); ^†††^C: controls; ^††††^Other health professionals: Nurses, Midwives, Optometrists and opticians, Physiotherapists and occupational therapists, Medical radiology technicians

Associations between selected occupations (ever occupied and by duration) and overall PCa are presented in Table 2. We observed positive associations between overall PCa risk and specific occupations lasting 10 years or more such as Surveyors, draughtsmen and related technicians (OR = 1.72 [1.06; 2.81]); Medical, dental and veterinary workers (OR = 3.75 [1.34; 10.49]), particularly for Medical doctors (OR = 3.98 [1.08; 14.72]); Administrative and managerial workers (OR = 1.72 [1.24; 2.38]), particularly for Legislative officials and Government administrators (OR = 2.88 [0.98; 8.41]) and Managers (OR = 1.62 [1.15; 2.27]); and Production supervisors and general foremen (OR = 1.78 [1.09; 2.91]). A positive association was also observed for men who had ever worked as Cabinetmakers and related woodworkers (OR = 2.94 [1.03; 8.43]).

However, we observed negative associations, for men who had ever worked as Service workers (OR = 0.78 [0.60; 1.01]) particularly for Building Caretakers, Charworkers, Cleaners and Related Workers (OR = 0.44 [0.21; 0.90]); Production and Related Workers, Transport Equipment Operators and Labourers (OR = 0.76 [0.60; 0.95]), particularly for Food and beverage processers (OR = 0.58 [0.35; 0.96]); and Transport Equipment Operators (OR = 0.73 [0.53; 0.99]).

Associations between selected occupations and PCa aggressiveness are presented in Table 3. We observed positive associations with aggressive PCa for men who had ever worked as Professional, technical and related workers (OR = 1.63 [1.11; 2.42]), especially for Surveyors, draughtsmen and related technicians (OR = 1.68 [1.00; 2.81]) and Medical, dental and veterinary workers (OR = 4.63 [1.33; 16.15]); and Fishermen, hunters and related workers (OR = 3.16 [1.00; 10.05]), particularly for Fishermen (OR = 4.01 [1.02; 15.69]. All those associations were particularly observed for a duration of employment of 10 years or more. Men who worked 10 years or more as Members of the armed forces also had an increased risk of aggressive PCa (OR = 5.14 [0.99; 26.71]), even though based on small numbers.

However, we did not observe negative associations with aggressive PCa in any occupational group.

## Discussion

Our results are based on a large population-based case-control study, carefully and specifically designed to address occupational factors and PCa risk, with a particular interest for aggressive PCa.

Our results showed an increased risk of PCa mainly among specific Professional, technical and related Workers and among Administrative and Managerial Workers, while we observed a decreased risk among Service workers and among Production and Related Workers, Transport Equipment Operators and Labourers.

Among the existing literature that has investigated occupational factors and PCa risk, very few studies assessed the entire occupational history, provided information on duration of employment in each occupation [14–16, 18]. Most of them studied PCa risk based on longest or last occupation held while only two studies took into account the aggressiveness of PCa [11, 15].

We observed positive associations with overall and aggressive PCa for men who ever worked as Surveyors, draughtsmen and related technicians, particularly if they worked 10 years or more. Similar results were also found in a North American study based on death certificates of men from 24 US states (OR = 2.10 [1.30; 3.50]), even though duration was not assessed [17].

We also found positive associations for Medical, dental and veterinary workers with the highest risk observed for Medical doctors. Our findings are consistent with previous cohort studies from Canada [5] and Nordic countries [7, 8] that showed a moderated increased risk for Medical doctors. However, they were not able to assess duration and prostate cancer aggressiveness. This “Medical, dental and veterinary workers” group is made up of men of a higher SES which may explain the observed positive associations based on a higher screening behaviour. In fact, the prevalence of screening in the last 2 years before interview among the Medical, Dental and veterinary workers group was 100%. However, our results were more specifically observed for aggressive PCa, thus minimizing a potential detection bias.

Working in Administrative and Managerial occupations for 10 years or more has been associated with an increased risk of PCa in our study, particularly for Legislative officials and Government administrators and Managers. These occupational categories are consistently observed to be associated with prostate cancer in the literature [5, 7, 8, 11, 14–16, 20].

While several studies found an increased risk of PCa with Protective service workers category [7, 8, 16, 32] and for Firefighters [6, 14, 16], Police officers [6, 9, 15] and Members of the armed forces [6–8, 11, 14], we did not observe any association with the Protective service workers category. However, we observed an increased risk of aggressive PCa in Members of the armed forces for men who worked 10 years or more even though based on small numbers.

When considering working in the Fishermen, Hunters and Related Workers group for 10 years or more, we observed an increased risk for aggressive PCa, particularly for Fishermen, while a decreased risk of either an early or a late-onset of PCa (whether diagnosed before or after 50 years old) for these workers was reported in the Nordic Occupational Cancer (NOCCA) studies [7, 8].

We found a positive association with PCa risk for Production Supervisors and General Foremen as observed in two North American case-control studies [16, 17].

Some negative associations have also been observed in our study with Service Workers with conflicting results in the literature [5, 13, 16] and with Food and Beverage Processers, Blacksmiths, Toolmakers and Machine-Tool Operators, Material-Handling and Related Equipment Operators, Dockers and Freight Handlers as observed in other original studies [5–8, 12].

Unlike some previous studies and reviews, we did not find any association in our study with Farmers and Agricultural industry [5, 6, 15, 16, 19, 21, 22, 24], Heavy and toxic metal workers or Chemical workers, Metalwork and Chemical industry [5, 7, 14, 18, 21, 22, 24].

Although we were able to study PCa risk in a wide range of occupations ever occupied and by duration of employment in each job, we could not clearly identify specific occupational exposures related to each job held, which may entail exposure to several different factors such as chemical, physical or psychosocial factors. We could not either rule out that certain of our results are chance findings due to multiple testing. However, we chose not to use any methods of correction for multiple testing. Indeed, we adopted an exploratory research with a hypothesis-generating purpose and we wanted to avoid reducing the power too drastically due to low already prevalence of certain occupations [33, 34]. We believed that our results could be used as leads for future research related to specific occupational exposures. Still and all, several factors in the work environment may explain the associations between specific occupations and PCa observed in our study.

Several studies on night-shift work and prostate cancer suggest that night and rotating shift work may be associated with prostate cancer risk [25, 26, 35], particularly for long duration and aggressive prostate cancer, including the EPICAP study [36, 37]. This may be relevant across some occupations identified in our study that are concerned by night-shift work, such as Medical, dental and veterinary workers, Members of the armed forces or Fishermen. Indeed, prevalence of night shift work in those occupations was 33.4% in Medical, dental and veterinary workers, 44.4% in Members of the armed forces, and 62.5% in Fishermen, Hunters and Related Workers. In addition, the three occupational groups mentioned before are also subject to a consistent chronic stress that may impact cancer development, as observed in a recent study that found a link between workplace stress and PCa risk [38]. According to the authors, chronic stress may influence cancer development by activating the sympathetic nervous system leading to downregulation of cellular immune response, genomic instability and changing in testosterone levels.

Furthermore, sedentary behaviour and low physical activity in the workplace may have a negative impact on PCa risk through changes in testosterone levels, insulin-like growth factor and immune function [39]. Occupations related to that in our study are Administrative and managerial occupations. Also, these workers usually have a higher SES than blue collar workers, which leads to better access to health services and an increase PSA screening behaviour [40, 41], which may explain part of our results, specifically observed for non-aggressive prostate cancer. In fact, the prevalence of screening in the last 2 years before interview was 93.4% among Managers and 90% among Legislative Officials and Government Administrators.

There is growing evidence that PCa development may be due to specific chemical exposures in some occupations. Based on the results of our study, we can mention at least two occupations that could be concerned: Members of the armed forces who are usually exposed to pesticides, solvents, fuels (diesel exhaust), chemical/warfare agents, particulate matter, polychlorinated biphenyls, polycyclic aromatic hydrocarbons (PAHs) and Woodworkers mainly exposed to wood dust, PAHs and pesticides [10, 42, 43]. However, several other occupations that may be associated with chemical exposures could also be related to PCa. Assessing chemical exposures using job exposure matrix will help to go further for studying occupational exposures in PCa.

Our findings are based on data from the EPICAP study which presents several strengths either in the selection of the population or in the data collection.

Cases were identified in all cancer hospitals, either public or private, that recruited prostate cancer patients in the department of Hérault. In 2011, 770 new cases of prostate cancer, of which 75% aged less than 75 years old, were reported by the Hérault Cancer Registry. Considering that the number of cases observed in 2012–2013 will be similar, approximately 1150 new cases were expected during the study period (2012–2013). In fact, the recruitment of cases was exhaustive since the number of eligible cases identified over the study period in the EPICAP study was 1098, thus limiting a potential selection bias. Moreover, even though participation rate in cases was 75%, the age distribution and the Gleason score of the non-respondent cases were comparable to those of the respondent cases, thus limiting a potential survival bias (private communication from the Hérault Cancer Registry). We were able to evaluate PCa risk taking into account aggressiveness, using the Gleason score, which has rarely been considered in previous occupational studies. Controls were randomly selected from the general population of the department of Hérault using quotas on age (5 years) to reflect the age distribution of the cases. Moreover, quotas by SES have been established in order for our control group to reflect to the general population of the department of Hérault of the same age. After the selection process, the distribution by SES between the control group and the male general population of the department of Hérault was compared and no significant difference was found, indicating that no major selection bias by SES had occurred in the control population.

Moreover, EPICAP is a population-based case-control study that has been specifically designed to study the role of environmental and occupational factors in PCa risk. We performed a standard lifetime work history that gathered information covering all jobs held more than 6 months throughout life. The job titles were derived from detailed information, such as starting and ending dates of each job held and specific tasks, provided by men about their entire employment history, this might have entailed errors. However, comparison between historical employment records and self-reported occupational questionnaires have generally shown a high concordance [44]. The coding of occupations have been performed by an industrial hygienist, blinded to the subject’s case/control status, thus reducing differential bias. Finally, we were able to consider the influence of duration of employment in each occupation on our results, by interval of 10 years because PCa latency can be very long, since lifetime occupational history was available for all subjects.

## Conclusions

We observed an increased risk of aggressive PCa for ≥10 years of employment among Medical, Dental and Veterinary Workers, Surveyors, draughtsmen and related technicians, Members of the armed forces and Fishermen, Hunters and Related Workers and a decreased of non-aggressive PCa risk for ≥10 years of employment among Production and Related Workers, Transport Equipment Operators and Labourers. These emerging associations can be used to lead future research investigating specific occupational exposures.

## Supplementary Information

**Additional file 1. **The results for associations between all occupations (1 to 3-digits with at least 10 subjects) and PCa can be found in a supplementary document named: Additional file 1 - Occupation and Prostate Cancer risk - results from the Epidemiological study of prostate cancer (EPICAP). Data are presented in a Microsoft Excel Spreadsheet (.xlsx) which contains: **Table S1.** Associations between occupations and prostate cancer [all cancers] (*N* = 1693). **Table S2.** Associations between occupations and prostate cancer aggressiveness (*N* = 1680).

## Data Availability

The datasets used and analysed during the current study are available from the corresponding author on reasonable request.

## Abbreviations

BMI: Body-mass index
EPICAP: Epidemiological study of prostate cancer
ISCO: International Standard Classification of Occupations
NOCCA: Nordic Occupational Cancer Study
OR: Odds ratios
PAHs: Polycyclic aromatic hydrocarbons
PCa: Prostate cancer
PSA: Prostatic specific antigen
SES: Socioeconomic status
95% CI: 95% confidence intervals

## References

[1] Grosclaude P, Belot A, Daubisse Marliac L, Remontet L, Leone N, Bossard N, Velten M, réseau Francim (2015). Prostate cancer incidence and mortality trends in France from 1980 to 2011. Prog Urol.

[2] Bray F, Ferlay J, Soerjomataram I, Siegel RL, Torre LA, Jemal A (2018). Global cancer statistics 2018: GLOBOCAN estimates of incidence and mortality worldwide for 36 cancers in 185 countries. CA Cancer J Clin.

[3] World Health Organization (2020). Global Health Observatory. Cancer today [Internet].

[4] Hsing AW, Devesa SS (2001). Trends and patterns of prostate Cancer: what do they suggest?. Epidemiol Rev.

[5] Sritharan J, MacLeod JS, McLeod CB, Peter A, Demers PA (2019). Prostate cancer risk by occupation in the occupational disease surveillance system (ODSS) in Ontario, Canada. Health Promot Chronic Dis Prev Can.

[6] Sritharan J, MacLeod J, Harris S, Cole DC, Harris A, Tjepkema M, Peters PA, Demers PA (2018). Prostate cancer surveillance by occupation and industry: the Canadian census health and environment cohort (CanCHEC). Cancer Med.

[7] Barry KH, Martinsen JI, Alavanja MCR, Andreotti G, Blair A, Hansen J, Kjærheim K, Koutros S, Lynge E, Sparèn P, Tryggvadottir L, Weiderpass E, Berndt SI, Pukkala E (2017). Risk of early-onset prostate cancer associated with occupation in the Nordic countries. Eur J Cancer.

[8] Pukkala E, Martinsen JI, Lynge E, Gunnarsdottir HK, Sparén P, Tryggvadottir L, Weiderpass E, Kjaerheim K (2009). Occupation and cancer - follow-up of 15 million people in five Nordic countries. Acta Oncol.

[9] Zeegers MPA, Friesema IHM, Goldbohm RA, van den Brandt PA (2004). A prospective study of occupation and prostate cancer risk. J Occup Environ Med.

[10] Koutros S, Beane Freeman LE, Lubin JH, Heltshe SL, Andreotti G, Barry KH, DellaValle CT, Hoppin JA, Sandler DP, Lynch CF, Blair A, Alavanja MCR (2013). Risk of Total and aggressive prostate Cancer and pesticide use in the agricultural health study. Am J Epidemiol.

[11] Adler C, Friesen MC, Yeboah ED, Tettey Y, Biritwum RB, Adjei AA, Tay E, Okyne V, Mensah JE, Truelove A, Yang B, Kelly SP, Zhou CK, McCullough LE, Pardo L, Hoover RN, Hsing AW, Cook MB, Koutros S (2019). Usual adult occupation and risk of prostate cancer in west African men: the Ghana prostate study. Occup Environ Med.

[12] Kaneko R, Zaitsu M, Sato Y, Kobayashi Y (2019). Risk of cancer and longest-held occupations in Japanese workers: a multicenter hospital-based case-control study. Cancer Med..

[13] Zaitsu M, Kaneko R, Takeuchi T, Sato Y, Kobayashi Y, Kawachi I (2019). Occupational class and male cancer incidence: Nationwide, multicenter, hospital-based case-control study in Japan. Cancer Med..

[14] Sritharan J, Demers PA, Harris SA, Cole DC, Peters CE (2017). Canadian Cancer registries epidemiology research group, et al. occupation and risk of prostate cancer in Canadian men: a case-control study across eight Canadian provinces. Cancer Epidemiol.

[15] Sauvé J-F, Lavoué J, Parent M-É (2016). Occupation, industry, and the risk of prostate cancer: a case-control study in Montréal. Can Environ Health.

[16] Krstev S, Baris D, Stewart P, Dosemeci M, Swanson GM, Greenberg RS, Schoenberg JB, Schwartz AG, Liff JM, Hayes RB (1998). Occupational risk factors and prostate cancer in U.S. blacks and whites. Am J Ind Med.

[17] Krstev S, Baris D, Stewart PA, Hayes RB, Blair A, Dosemeci M (1998). Risk for prostate cancer by occupation and industry: a 24-state death certificate study. Am J Ind Med.

[18] Aronson KJ, Siemiatycki J, Dewar R, Gérin M (1996). Occupational risk factors for prostate cancer: results from a case-control study in Montréal, Québec. Can Am J Epidemiol.

[19] van der Gulden JW, Kolk JJ, Verbeek AL (1995). Work environment and prostate cancer risk. Prostate..

[20] Pearce NE, Sheppard RA, Fraser J (1987). Case-control study of occupation and cancer of the prostate in New Zealand. J Epidemiol Community Health.

[21] Parent M-E, Siemiatycki J (2001). Occupation and prostate Cancer. Epidemiol Rev.

[22] Doolan G, Benke G, Giles G (2014). An update on occupation and prostate cancer. Asian Pac J Cancer Prev.

[23] Wendeu-Foyet MG, Menegaux F (2017). Circadian disruption and prostate Cancer risk: an updated review of epidemiological evidences. Cancer Epidemiol Biomark Prev.

[24] Krstev S, Knutsson A (2019). Occupational risk factors for prostate Cancer: a Meta-analysis. J Cancer Prev.

[25] Rao D, Yu H, Bai Y, Zheng X, Xie L (2015). Does night-shift work increase the risk of prostate cancer? A systematic review and meta-analysis. Oncol Targets Ther.

[26] IARC Monographs Vol 124 group (2019). Carcinogenicity of night shift work. Lancet Oncol.

[27] Straif K, Baan R, Grosse Y, Secretan B, El Ghissassi F, Bouvard V, et al. Carcinogenicity of shift-work, painting, and firefighting.Lancet Oncol. 2007;8(12):1065–6. 10.1016/S1470-2045(07)70373-X. 10.1016/S1470-2045(07)70373-X 19271347

[28] Baan R, Grosse Y, Straif K, Secretan B, El Ghissassi F, Bouvard V, et al. A review of human carcinogens--Part F: chemicalagents and related occupations. Lancet Oncol. 2009;10(12):1143–4. 10.1016/s1470-2045(09)70358-4. 10.1016/s1470-2045(09)70358-4 19998521

[29] Menegaux F, Anger A, Randrianasolo H, Mulot C, Laurent-Puig P, Iborra F (2014). Epidemiological study of prostate cancer (EPICAP): a population-based case–control study in France. BMC Cancer.

[30] International Labour Organisation. International standard classification of occupations. Revised, 1968 [Internet]. Geneva:ILO; 1968. Available from: https://ilo.primo.exlibrisgroup.com/permalink/41ILO_INST/j3q9on/alma991141423402676.

[31] Egevad L, Granfors T, Karlberg L, Bergh A, Stattin P (2002). Prognostic value of the Gleason score in prostate cancer. BJU Int.

[32] Harris MA, Kirkham TL, MacLeod JS, Tjepkema M, Peters PA, Demers PA (2018). Surveillance of cancer risks for firefighters, police, and armed forces among men in a Canadian census cohort. Am J Ind Med.

[33] Rothman KJ (1990). No adjustments are needed for multiple comparisons. Epidemiology..

[34] Labrèche F, Goldberg MS, Valois M-F, Nadon L (2010). Postmenopausal breast cancer and occupational exposures. Occup Environ Med.

[35] Gan Y, Li L, Zhang L, Yan S, Gao C, Hu S, Qiao Y, Tang S, Wang C, Lu Z (2018). Association between shift work and risk of prostate cancer: a systematic review and meta-analysis of observational studies. Carcinogenesis..

[36] Wendeu-Foyet MG, Bayon V, Cénée S, Trétarre B, Rébillard X, Cancel-Tassin G, Cussenot O, Lamy PJ, Faraut B, Ben Khedher S, Léger D, Menegaux F (2018). Night work and prostate cancer risk: results from the EPICAP study. Occup Environ Med.

[37] Papantoniou K, Castaño-Vinyals G, Espinosa A, Aragonés N, Pérez-Gómez B, Burgos J, Gómez-Acebo I, Llorca J, Peiró R, Jimenez-Moleón JJ, Arredondo F, Tardón A, Pollan M, Kogevinas M (2015). Night shift work, chronotype and prostate cancer risk in the MCC-Spain case-control study. Int J Cancer.

[38] Blanc-Lapierre A, Rousseau M-C, Parent M-E (2017). Perceived Workplace Stress Is Associated with an Increased Risk of Prostate Cancer before Age 65. Front Oncol [Internet].

[39] Liu Y, Hu F, Li D, Wang F, Zhu L, Chen W, Ge J, An RH, Zhao YS (2011). Does physical activity reduce the risk of prostate cancer? A systematic review and meta-analysis. Eur Urol.

[40] Kilpeläinen TP, Talala K, Raitanen J, Taari K, Kujala P, Tammela TLJ, Auvinen A (2016). Prostate Cancer and socioeconomic status in the Finnish randomized study of screening for prostate Cancer. Am J Epidemiol.

[41] Liu L, Cozen W, Bernstein L, Ross RK, Deapen D (2001). Changing relationship between socioeconomic status and prostate cancer incidence. J Natl Cancer Inst.

[42] Siemiatycki J, Richardson L, Straif K, Latreille B, Lakhani R, Campbell S, Rousseau MC, Boffetta P (2004). Listing occupational carcinogens. Environ Health Perspect.

[43] US Department of Veterans Affairs, Veterans Health (2020). Military Exposures - Public Health [Internet].

[44] Teschke K, Olshan AF, Daniels JL, De Roos AJ, Parks CG, Schulz M (2002). Occupational exposure assessment in case-control studies: opportunities for improvement. Occup Environ Med.

